# Risks and clinical predictors of cirrhosis and hepatocellular carcinoma diagnoses in adults with diagnosed NAFLD: real-world study of 18 million patients in four European cohorts

**DOI:** 10.1186/s12916-019-1321-x

**Published:** 2019-05-20

**Authors:** Myriam Alexander, A. Katrina Loomis, Johan van der Lei, Talita Duarte-Salles, Daniel Prieto-Alhambra, David Ansell, Alessandro Pasqua, Francesco Lapi, Peter Rijnbeek, Mees Mosseveld, Dawn M. Waterworth, Stuart Kendrick, Naveed Sattar, William Alazawi

**Affiliations:** 1 Real World Data, GlaxoSmithKline, Uxbridge, UK; 2 Worldwide Research and Development, Pfizer, Genome Sciences and Technologies, New York, USA; 3 Erasmus Universitair Medisch Centrum, Rotterdam, Netherlands; 4 Fundació Institut Universitari per a la Recerca a l’Atenció Primària de Salut Jordi Gol i Gurina, Barcelona, Spain; 5 Centre for Statistics in Medicine, NDORMS, University of Oxford, Oxford, UK; 6 Quintile IMS, London, UK; 7 Health Search, Italian College of General Practitioners and Primary Care, Firenze, Italy; 8 Genetics, GlaxoSmithKline, Collegeville, PA USA; 9 GlaxoSmithKline, Medicines Research Centre, Cambridge, UK; 10 University of Glasgow, Glasgow, UK; 11 Barts Liver Centre, Blizard Institute, Queen Mary, University of London, London, UK

**Keywords:** Cirrhosis, Hepatocellular cancer, NAFLD, NASH, Population

## Abstract

**Background:**

Non-alcoholic fatty liver disease (NAFLD) is a common condition that progresses in some patients to steatohepatitis (NASH), cirrhosis and hepatocellular carcinoma (HCC). Here we used healthcare records of 18 million adults to estimate risk of acquiring advanced liver disease diagnoses in patients with NAFLD or NASH compared to individually matched controls.

**Methods:**

Data were extracted from four European primary care databases representing the UK, Netherlands, Italy and Spain. Patients with a recorded diagnosis of NAFLD or NASH (NAFLD/NASH) were followed up for incident cirrhosis and HCC diagnoses. Each coded NAFLD/NASH patient was matched to up to 100 “non-NAFLD” patients by practice site, gender, age ± 5 years and visit recorded within ± 6 months. Hazard ratios (HR) were estimated using Cox models adjusted for age and smoking status and pooled across databases by random effects meta-analyses.

**Results:**

Out of 18,782,281 adults, we identified 136,703 patients with coded NAFLD/NASH. Coded NAFLD/NASH patients were more likely to have diabetes, hypertension and obesity than matched controls. HR for cirrhosis in patients compared to controls was 4.73 (95% CI 2.43–9.19) and for HCC, 3.51 (95% CI 1.72–7.16). HR for either outcome was higher in patients with NASH and those with high-risk Fib-4 scores. The strongest independent predictor of a diagnosis of HCC or cirrhosis was baseline diagnosis of diabetes.

**Conclusions:**

Real-world population data show that recorded diagnosis of NAFLD/NASH increases risk of life-threatening liver outcomes. Diabetes is an independent predictor of advanced liver disease diagnosis, emphasising the need to identify specific groups of patients at highest risk.

**Electronic supplementary material:**

The online version of this article (10.1186/s12916-019-1321-x) contains supplementary material, which is available to authorized users.

## Background

Non-alcoholic fatty liver disease (NAFLD) is the most common cause of liver disease worldwide. NAFLD represents a spectrum of disease that includes simple steatosis, non-alcoholic steatohepatitis (NASH) and fibrosis [1]. The numbers of individuals presenting with end-stage complications of NASH, namely decompensated cirrhosis and hepatocellular carcinoma (HCC), are rising [2, 3], and NASH is rapidly becoming the most common indication for liver transplantation [4]. Yet not all patients within the NAFLD spectrum progress, and for the majority, NAFLD is a benign condition [1]. A key clinical challenge is to identify the proportion of patients who are at high risk of developing advanced liver disease, so that interventions, including the many novel therapies in development, can be targeted to those at greatest need.

Our current understanding of NAFLD epidemiology and progression largely derives from single-centre studies of small- or medium-sized cohorts and meta-analyses of these [5–7]. These studies, together with emerging data from placebo arms of therapeutic trials [8], have taught us that patients with existing evidence of progressive disease (e.g., fibrosis) are at risk of further progression to HCC and decompensated cirrhosis, albeit this may reflect a degree of lead-time bias. Such studies often involve formal assessment of well-phenotyped patients at inclusion but are, by design, selective and may not represent the ‘real-world’ situation for the majority of patients with NAFLD. Paired biopsy data have been reported, although the second biopsy is often performed because of clinical suspicion and not per study protocol, which may bias estimates of progression [9]. Real-world patients are socially and ethnically diverse, have comorbidities and concomitant medications or simply cannot commit to long-term studies or trials and therefore may not be represented by any of these study designs.

Increasingly, real-world data derived from primary care electronic health records (EHR) of a sizeable proportion of the general population [10, 11] are being used to address these issues. In many European countries, where healthcare is largely state-funded and there are low or absent primary care co-payments, the population has unrestricted access to healthcare via primary care physicians who act as gatekeepers for referral to secondary care [12]. People register with primary care centres at birth or when they move to an area in order to access healthcare; therefore, primary care EHR represent data that are as close to the ‘general’ population as possible. If a practice joins the database, all the patients at that practice are registered in the database and, although there is an option for individual patients to opt out, this is minimal (< 1%).

In order to gain insights into the NAFLD spectrum of diseases in real-world patients, we extracted data from four large European primary care databases and identified a cohort of patients with a diagnosis of NAFLD or of NASH. Our aim in this study was to estimate the risk for patients with diagnoses of NAFLD or NASH to acquire a new diagnosis of cirrhosis and HCC and to understand the main predictors for this.

## Methods

### Databases

Databases were accessed via the European Medical Information Framework (EMIF) network: The Health Search Database (HSD) in Italy [13], The Integrated Primary Care Information (IPCI) in the Netherlands [14], the Information System for the Development of Research in Primary Care (SIDIAP) in Spain [15] and The Health Information Network (THIN) in the UK [16] (Additional file 1: Table S1). HSD collects electronic medical record data from a network of over 800 Italian GPs who are members of the Italian College of General Practitioners. IPCI is a longitudinal collection of electronic patient records from over 750 Dutch general practitioners, containing data from over 2 million patients. SIDIAP collects data from 274 primary care practices comprising 3414 basic care units [17], and THIN contains the electronic medical records of 11.1 million patients from 562 general practices in the UK, covering 6.2% of the UK population [18]. The data custodians for each database provided approval that the protocol of the study complied with local privacy laws. Anonymised data were extracted locally by each data custodian liaising with the EMIF Platform and using a data transformation tool called Jerboa Reloaded [10]. The data were then uploaded onto a secure remote server maintained by an independent academic centre (Erasmus Medical Centre Private Research Environment, Netherlands) and analysed centrally.

### Study design

We conducted a matched cohort study. All patients with a diagnosis of NAFLD or NASH (termed NAFLD/NASH) prior to 01/01/2016 were identified in the four databases using harmonisation methods previously described [10]. Patients were included in the analysis if they were aged ≥ 18 at diagnosis and had medical records available for ≥ 12 months from registration with the practice. Exclusion criteria were missing information on age and sex, a record of alcohol abuse at any time prior to diagnosis and a history of liver morbidity within the 12 months prior to diagnosis [10] (see Additional file 1: Supplementary Methods for exclusion diagnoses).

Each NAFLD/NASH patient was matched with up to 100 ‘non-exposed’ controls who did not have a NAFLD or NASH diagnosis at or prior to the index date (defined as the date of diagnosis of the matched NAFLD/NASH patient). Matching was done by practice site, age at index date ± 5 years, sex and a visit at the practice within ± 6 months of the index date.

In the THIN and SIDIAP databases, the terminology of the database (Read code and International Classification of Disease version 10, ICD10, respectively) allowed NAFLD and NASH diagnoses to be distinguished from each other. Therefore, in these databases, a matched control cohort was constructed for each of the diagnoses: NAFLD, NASH and, to enable comparison between all databases, NAFLD/NASH. If a patient had both NAFLD and NASH diagnoses recorded, the earliest event was used to define index date of NAFLD/NASH diagnosis, and the NASH diagnosis deemed an incident event. In HSD (ICD 9) and IPCI (IPCI Dutch), where NAFLD and NASH could not be distinguished, only one cohort (NAFLD/NASH) was defined and controls matched to this.

Patients were followed up from the index date until the earliest of occurrence of cirrhosis, hepatocellular carcinoma or NASH (where this could be identified), end of the study period (31/12/2015) and loss of follow-up due to exit out of the database or death. Events of interest were incident diagnosis of cirrhosis, hepatocellular carcinoma or NASH, where this could be identified. See Additional file 1: Supplementary Methods for variable extraction and data analysis.

## Results

Out of 18,782,281 eligible individuals in the four databases, we identified 136,703 (0.7%) who had a recorded diagnosis of either NAFLD or NASH (coded NAFLD/NASH) and who met the inclusion criteria (Additional file 1: Table S1). The Spanish (SIDIAP) and UK (THIN) databases contributed 71% of all cases; the remaining 29% of coded NAFLD/NASH cases were from the Dutch (IPCI) and Italian (HSD) databases. In SIDIAP, 2.5% of all coded NAFLD/NASH patients (*n* = 1880) had NASH, and in THIN, this was 4.7% (*n* = 1212). Due to the coding, NAFLD and NASH could not be distinguished in IPCI and HSD. Therefore, in the initial phase of analysis, we combined all NAFLD and NASH codes from all four databases as coded NAFLD/NASH.

Comparing coded NAFLD/NASH patients across the four databases, there were minor differences between databases in mean age, BMI and proportion with diabetes (Table 1 and Additional file 1: Table S2). BMI data were available in 64.6% of patients with coded NAFLD/NASH and in 45.9% of matched controls (Additional file 1: Table S3). In the subset of patients for whom data were available, ALT and AST values were highest in THIN, and the proportion of obese patients highest in SIDIAP. Sufficient data were available to calculate the non-invasive fibrosis Fib-4 score (age, AST, ALT and platelets) in 46.7% of patients (range 12.6–62.6%, Table 2). THIN (UK) had the smallest proportion of patients with Fib-4 data (12.6%), in whom the proportion of patients with high-risk scores was 10.5%, highest among the four databases.

**Table 1:** Descriptive characteristics of coded NAFLD/NASH patients and matched unexposed cohorts

Baseline characteristics	HSD - Italy		IPCI - UK		SIDIAP - Spain		THIN - UK		Total population	
	NAFLD/NASH	Matched non-NAFLD/NASH	NAFLD/NASH	Matched non-NAFLD/NASH	NAFLD/NASH	Matched non-NAFLD/NASH	NAFLD/NASH	Matched non-NAFLD/NASH	NAFLD/NASH	Matched non-NAFLD/NASH
Follow-up years prior to index date: median (IQR)	7.5 (4.66–10.5)	7.7 (4.8–10.6)	2.2 (0.9–3.8)	2.2 (1–3.8)	5.2 (3.1–7.1)	5.2 (3.1–7.1)	13.4 (5.5–23.4)	13.9 (5.8–23.4)	5.4 (2.8–8.0)	5.5 (2.9–8.3)
Follow-up years post index date: median (IQR)	5.3 (2.7–8.1)	5.2 (2.7–7.9)	1.8 (0.8–3.2)	1.8 (0.8–3.3)	3.5 (1.7–5.6)	3.5 (1.7–5.7)	3.1 (1.3–5.9)	3.0 (1.3–5.7)	3.3 (1.5–5.8)	3.2 (1.5–5.7)
Age in years, mean (SD)	56.1 (14.4)	55.0 (13.7)	56.8 (13.9)	56.1 (13.5)	55.9 (13.4)	54.5 (13.1)	54.4 (13.4)	53.5 (13.5)	55.8 (13.6)	54.6 (13.3)
Gender, % of Males	57.3	54.7	49.2	48.5	52.6	48.8	51.5	51.0	52.7	50.1
Current smokers*, %	11.3	9.0	17.2	11.5	17.6	15.4	17.8	19.0	16.5	14.4
Body mass index in kg/m^2^, mean (SD)	29.8 (5.0)	27.5 (5.0)	30.8 (5.3)	28.3 (5.2)	31.4 (5.1)	28.7 (5.1)	32.5 (6.0)	28.5 (5.9)	31.3 (5.3)	28.5 (5.3)
Obesity (%)	21.5	9.0	23.6	9.0	39.5	18.1	49.6	18.2	36.0	15.4
History of type 2 diabetes (%)	17.6	11.0	20.4	9.2	19.9	10	21.5	7.1	19.8	9.6
History of hypertension (%)	47.2	36.6	36.0	26.2	42.6	29.3	41.2	25.9	42.2	29.6
Statin use (%)	22.0	15.8	33.2	22.0	6.9	6.8	32.6	18.5	16.9	12.1
Aspartate transaminase (IU/L), median (IQR)	24 (19–32)	20 (17–25)	29 (22–40)	23 (20–28)	29 (22–40)	22 (18–27)	31 (23–45)	22 (19–27)	28 (21–39)	21 (18–26)
Alanine transaminase (IU/L), median (IQR)	30 (20–48)	21 (16–29)	37 (25–55)	25 (18–33)	34 (22–53)	20 (15–28)	42 (26–65)	22 (17–31)	35 (23–54)	21 (16–29)

*After imputation of missing as non-smokers. *N* number of individuals. For laboratory values, we exclude outlier values greater than mean + 3 × SD (mean and SD computed separately in NAFLD and non-NAFLD separately)

**Table 2:** Distribution of Fib-4 scores in coded NAFLD/NASH patients shown for each country database

Risk category	HSD Italy (%)	IPCI Netherlands (%)	SIDIAP Spain (%)	THIN UK (%)	Total population (%)
Low (< 1.30)	64.7	69.7	65.3	63.4	65.4
Indeterminate (1.30–2.67)	31.0	26.6	30.1	26.2	29.8
High (> 2.67)	4.3	3.7	4.6	10.5	4.7

Patients with a coded diagnosis of NAFLD/NASH had comparable age and sex distribution, smoking rates and duration of follow-up as matched controls (Table 1). As expected, however, controls had lower BMI; lower rates of obesity, hypertension or diabetes; and lower serum levels of ALT and AST.

### Risk of incident cirrhosis and HCC is higher in NAFLD/NASH patients compared to controls

**Fig. 1:**
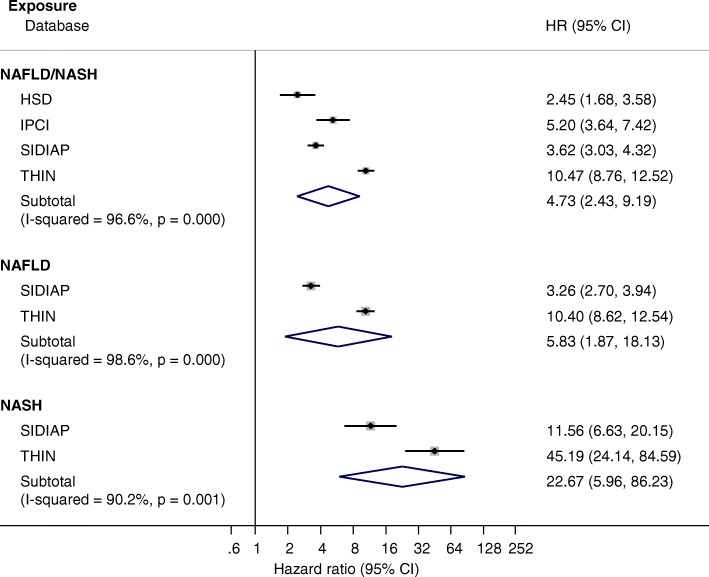
Association of coded NAFLD/NASH, NAFLD and NASH with cirrhosis. Hazard ratios and 95% confidence interval for acquiring a new diagnosis of cirrhosis in each database and combined across databases (subtotal)

Combining all four databases, the median duration of follow-up was 3.3 years (IQR 1.8–5.3) totalling 531,452 person-years for patients with coded NAFLD/NASH and 43,385,495 person-years for controls. Among all coded NAFLD/NASH patients, the incidence of cirrhosis diagnosis was 0.76 per 1000 person-years, (95% confidence interval (CI) 0.46 to 2.32), and the incidence of hepatocellular carcinoma diagnosis was 0.3 per 1000 person-years, (0.26 to 0.60; Additional file 1: Table S4). Patients with coded NAFLD/NASH were at significantly higher risk of acquiring a new diagnosis of cirrhosis compared to controls with a pooled HR of 4.73 (95%CI 2.43–9.19) after adjustment for age, smoking status and BMI (Fig. 1).

**Fig. 2:**
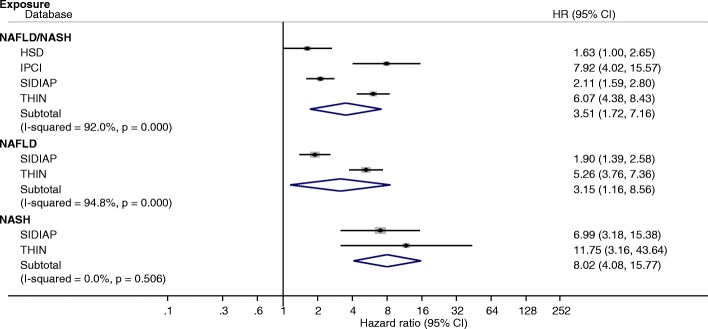
Association of coded NAFLD/NASH, NAFLD and NASH with hepatocellular carcinoma (HCC). Hazard ratios and 95% confidence interval for acquiring a new diagnosis of HCC in each database and combined across databases (subtotal)

Similarly, the risk of incident HCC diagnosis was significantly higher in coded NAFLD/NASH patients compared to controls. The pooled HR across the four databases for an incident diagnosis of HCC was 3.51 (95%CI 1.72–7.16 Fig. 2). There were no significant differences in the HRs when categorising patients into those with and without obesity, smoking, diabetes or hypertension; male sex and older age (Additional file 1: Figure S1). There were no significant differences in the HRs for cirrhosis and HCC diagnoses following adjustment for age and smoking alone in all coded NAFLD/NASH patients compared to patients with available BMI data (Additional file 1: Figures S2 and S3). This is despite the fact that patients with BMI data were more likely to be smokers (19.5% vs 11.2%), diabetic (26.9% vs 7.0%) and hypertensive (50.1% vs 27.9%, Additional file 1: Table S5).

### Fib-4 predicts disease progression in patients with NAFLD/NASH

In the subset of coded NAFLD/NASH patients in whom we could calculate Fib-4 (*n* = 63,971, Additional file 1: Table S3), the incidence of a new diagnosis of cirrhosis was significantly higher for the high-risk compared to low-risk category (HR 33.24, 95%CI 8.82–125.34), adjusting for age and smoking status and more modest, albeit still significant, for the intermediate compared to low-risk group (HR 5.04, 95%CI 2.30–11.04 Additional file 1: Figure S4A). Similarly, compared to patients with low-risk scores, the incidence of an HCC diagnosis was higher in patients with indeterminate (HR 3.74, 95%CI 1.76–7.96) or high-risk scores (HR 25.2, 95%CI 7.83–80.66, Additional file 1: Figure S4B).

### Distinguishing NAFLD from NASH diagnoses when estimating risk of cirrhosis and HCC

The pooled HR for incident NASH diagnosis in patients with a coded diagnosis of NAFLD compared to controls was 7.75 (95%CI 2.56–23.51, *p* = 0.008) although this estimate is based on a very small number of individuals (*n* = 130 of whom only seven were in SIDIAP, Additional file 1: Figure S5). In the subset of patients with a coded diagnosis of NASH, the incidence of diagnoses of liver outcomes was higher than in those with NAFLD albeit confidence intervals overlapped: 3.25 per 1000 person-years (95%CI 2.41–4.10) for cirrhosis and 1.16 per 1000 person-years (95%CI 0.67–1.65) for HCC (Figs. 1 and 2).

### Short time interval to cirrhosis diagnosis in patients with NAFLD and NASH

In SIDIAP, 174 out of 75,415 patients with coded NAFLD were coded as having cirrhosis (incidence rate 0.66 per 1000 person-years (95%CI 0.56–0.76) with a median time to the new diagnosis of 2.9 years whereas 38 out of 1880 patients with NASH acquired a diagnosis of cirrhosis (incidence rate 2.83 per 1000 person-years (95%CI 2.0–3.88, Additional file 1: Table S4) with a similar median time to diagnosis of 3.0 years (Additional file 1: Table S6). In THIN, the incidence of cirrhosis was higher and the interval between diagnoses was shorter for both stages of disease. One hundred three out of 24,743 patients with coded NAFLD acquired a cirrhosis diagnostic code (incidence rate 2.17 per 1000 person-years (95%CI 1.86–2.51) with median time to diagnosis of 2.0 years, compared to 26 out of 1212 patients with coded NASH (incidence rate 5.81 per 1000 person-years (95% CI 3.8–8.52) with median time to diagnosis of 0.5 years.

### Diabetes predicts disease progression

In coded NAFLD/NASH patients, the strongest association with incident liver outcomes was observed in patients who also had a past diagnosis of diabetes at baseline (HR 2.3, 95% CI 1.9–2.78). In matched controls without coded NAFLD/NASH, smoking was also associated with liver outcome (HR 1.5, 95% CI 1.41–1.6) in addition to the independent risk attributed to diabetes, which was higher than in patients with coded NAFLD/NASH (HR 2.92, 95% CI 2.76–3.08, Table 3).

**Table 3:** Association between covariates and risk of liver outcomes: cirrhosis or hepatocellular carcinoma. Using a 1-step Cox model stratified by database

	NAFLD/NASH HR (95% CI)	Matched control HR (95% CI)
Smoking status (current/not current)	1.19 (0.94; 1.51)	1.50 (1.41; 1.60)
Age (years)	1.04 (1.03; 1.05)	1.04 (1.03; 1.04)
History of diabetes (yes/no)	2.30 (1.90; 2.78)	2.92 (2.76; 3.08)
History of hypertension (yes/no)	0.92 (0.76; 1.12)	1.07 (1.01; 1.13)
BMI (kg/m^2^)	1.01 (1.00; 1.03)	1.04 (1.03; 1.04)

## Discussion

To our knowledge, this is the largest study to date that has used EHR data to investigate rates of new diagnoses of advanced liver disease in patients with NAFLD. Our patients were well-matched to a very large number of controls according to sex, age, GP practice and most recent visit, thus limiting bias due to geographical and socioeconomic diversity and behaviours relating to health service utilisation. Patients with coded NAFLD/NASH are at significantly increased risk of acquiring a diagnosis of cirrhosis or HCC, compared to matched controls. The risk is greater in patients with a coded diagnosis of NASH compared to NAFLD and in those with high-risk Fib-4 fibrosis scores compared to indeterminate or low-risk scores. Diabetes is an independent risk factor for progression to either HCC or cirrhosis diagnoses in both coded NAFLD/NASH patients and matched controls.

We applied minimal selection criteria and therefore were able to include over 78% of all adults registered in the databases, hence the ‘real-world’ nature of the study. The overall proportion of people with coded NAFLD/NASH diagnoses is lower than expected as reported previously [10], is in keeping with other primary care work [19] and may reflect levels of awareness of NAFLD/NASH in primary care [20, 21]. Hence, our data, by definition, can only represent the visible part of the clinical iceberg. Despite this, we find that patients with coded NAFLD/NASH acquire diagnoses of life-threatening liver disease within a relatively short follow-up period (median 3.3 years).

It is not feasible that the short time intervals between coded diagnosis of NAFLD/NASH and advanced liver disease reflect true rates of disease progression, estimated to be one fibrosis stage per 7 years [22]. The acquisition of a new code in the healthcare record does not necessarily mean that pathological progression has occurred at that time, nor that the stage did not exist at baseline. Our interpretation of these data is that patients in Europe are being diagnosed at the later stages of disease, which are associated with greater risk of liver-related mortality [23–25].

Less than 50% of patients had sufficient data to calculate Fib-4, the components of which are also needed to calculate many other non-invasive fibrosis scores [26]. There was marked national variation in fibrosis assessment; 73.1% of patients in whom we could calculate Fib-4 were from the Spanish database. We have no way of determining whether these scores were actually calculated by clinicians and whether they influenced decision-making. This is despite the fact that such risk stratification is central to most guidelines [27–29], used to determine clinical management, select patients for clinical trials and probably triage patients for future therapy.

In the databases where NAFLD/NASH codes could not be distinguished (HSD and IPCI), even those with low-risk Fib-4 scores were at increased risk of cirrhosis and HCC compared to controls. This further suggests that primary care records under-estimate disease severity and that some patients with NAFLD/NASH diagnoses actually have advanced fibrosis or cirrhosis already. Apart from a diagnosis of NAFLD/NASH, diabetes was the strongest independent risk factor for acquiring a diagnosis of cirrhosis or HCC. In the matched control population, the HR for diabetes was even higher than the coded NAFLD/NASH cohort, which may reflect a significant number of individuals with undiagnosed NAFLD/NASH among the controls. The importance of diabetes is consistent with a review of patients who had undergone more than one biopsy in the course of their routine clinical care in the UK, which showed that diabetes was a risk factor for progression of fibrosis [9]. Obesity is an important risk factor for many cancers including HCC [30], but we did not find that in our study. If patients are diagnosed late in the disease spectrum, it is unlikely that patients will have undergone surveillance and HCC may be diagnosed at late stages when symptoms including weight loss are manifest. Taken together, these findings emphasise the need to recognise risk factors for progressive disease and to detect disease at early stages when interventions can be more effective.

This study is subject to limitations. The nature of real-world data is such that we cannot ascertain the origin of codes nor the motivation for adding diagnoses to the patient record. Although the study is based in primary care, it is likely that a large proportion of diagnoses will have been made with some involvement of secondary care. It would be inaccurate to assume that all patients who carry the code ‘NASH’ have had a liver biopsy and histological assessment and it might be that the diagnosis was assumed and recorded based on, for example, ultrasound evidence of fatty liver and elevated serum transaminases or increased stiffness on transient elastography. Similarly, it was not possible to confirm that the matched controls did not have NAFLD/NASH. However, the clinical features of patients with coded NAFLD/NASH are consistent with the diagnostic codes, although if patients with NAFLD/NASH do exist in the control group then the effect sizes reported here are underestimates of the real risk. This means that there are individuals living with diabetes in primary care who have not been diagnosed with NAFLD/NASH but are at significantly increased risk of developing liver cirrhosis and cancer.

The estimated size of the NAFLD problem has raised fears of large unmanageable patient numbers who are not at immediate threat of disease. Notwithstanding our expectation that many cases have not been identified in this study, we have shown that 0.6% of patients with an existing coded diagnosis of NAFLD/NASH acquire a diagnosis of cirrhosis and/or HCC within a 3-year follow-up period. This gives us insight into the rate at which advanced disease is discovered, even if this is not the natural history in the general population. The clinical impact of our data is that they highlight the large gaps in diagnosis and risk assessment of NAFLD and NASH with variable rates of risk stratification, staging of disease and seemingly late diagnosis.

## Conclusions

Our knowledge of NAFLD/NASH is being based on small, highly selected cohort studies. These have been accurate in telling us the potential scale of the prevalence and progression of disease, but the reality for many in the general population is some way from that. In order to affect population health and make an impact on the overall health burden of advanced liver disease, we cannot simply rely on introducing effective therapies to the small number of people with established diagnoses. The current approach to opportunistically investigate those in whom abnormalities in liver tests arise is clearly not working. While better biomarkers are needed that identify those at risk more precisely, the current tools are not being used, leaving many patients unclear as to the stage of their disease and its significance to their health. Therefore, making an impact on advanced liver disease will need co-ordinated efforts to identify those with NAFLD, to stage their disease and target those at risk of progression.

## Additional file

Additional file 1: Supplementary Methods. **Table S1.** Attrition table showing patients with recorded diagnoses of NAFLD or NASH and matched unexposed controls. **Table S2.** Descriptive characteristics of coded NAFLD or NASH patients and matched unexposed cohorts in SIDIAP and THIN. **Table S3.** Number of patients with data available in coded NAFLD/NASH and matched unexposed cohorts. **Table S4.** Incidence rate of liver outcomes in four primary care databases. **Table S5.** Descriptive characteristics of coded NAFLD/NASH patients and matched non-NAFLD/NASH in a sample of patients with and without BMI data available, all datasets combined. **Table S6.** Median and interquartile range (in years) for time to event in coded NAFLD and matched non-NAFLD who experience a cirrhosis or hepatocellular carcinoma event during follow-up. **Figure S1.** Subgroup analysis of the association between coded NAFLD/NASH and incident (A) cirrhosis and (B) hepatocellular carcinoma events by medical history and demographics. **Figure S2.** Hazard ratio (HR) for cirrhosis (A) adjusted for age and smoking in all patients and (B) adjusted for age and smoking in patients with BMI. **Figure S3.** Hazard ratio (HR) for HCC (A) adjusted for age and smoking in all patients and (B) adjusted for age and smoking in patients with BMI. **Figure S4.** Fib-4 Association with (A) cirrhosis or (B) HCC. **Figure S5.** Risk of coded NASH in patients with coded NAFLD. (DOCX 540 kb)

## Abbreviations

ALT: Alanine transaminase
AST: Aspartate transaminase
BMI: Body mass index
CI: Confidence interval
EHR: Electronic Health Record
EMIF: European Medical Information Framework
GP: General Practitioner
HCC: Hepatocellular carcinoma
HSD: Health Search Database
IPCI: Information System for Research in Primary Care
LFT: Liver function tests
NAFLD: Non-alcoholic fatty liver disease
NASH: Non-alcoholic steatohepatitis
SIDIAP: Information System for Research in Primary Care
THIN: The Health Improvement Network
UK: United Kingdom
US: United States
