# Supplementary material for: Risks and clinical predictors of cirrhosis and hepatocellular carcinoma diagnoses in adults with diagnosed NAFLD: real-world study of 18 million patients in four European cohorts
Source: BMC Med. 2019 May 20;17:95. doi: 10.1186/s12916-019-1321-x (PMC6526616; doi:10.1186/s12916-019-1321-x)
Supplement: Supplementary file 1 — Supplementary Methods. Table S1. Attrition table showing patients with recorded diagnoses of NAFLD or NASH and matched unexposed controls. Table S2. Descriptive characteristics of coded NAFLD or NASH patients and matched unexposed cohorts in SIDIAP and THIN. Table S3. Number of patients with data available in coded NAFLD/NASH and matched unexposed cohorts. Table S4. Incidence rate of liver outcomes in four primary care databases. Table S5. Descriptive characteristics of coded NAFLD/NASH patients and matched non-NAFLD/NASH in a sample of patients with and without BMI data available, all datasets combined. Table S6. Median and interquartile range (in years) for time to event in coded NAFLD and matched non-NAFLD who experience a cirrhosis or hepatocellular carcinoma event during follow-up. Figure S1. Subgroup analysis of the association between coded NAFLD/NASH and incident (A) cirrhosis and (B) hepatocellular carcinoma events by medical history and demographics. Figure S2. Hazard ratio (HR) for cirrhosis (A) adjusted for age and smoking in all patients and (B) adjusted for age and smoking in patients with BMI. Figure S3. Hazard ratio (HR) for HCC (A) adjusted for age and smoking in all patients and (B) adjusted for age and smoking in patients with BMI. Figure S4. Fib-4 Association with (A) cirrhosis or (B) HCC. Figure S5. Risk of coded NASH in patients with coded NAFLD. (DOCX 540 kb) [file 12916_2019_1321_MOESM1_ESM.docx]

**Supplementary Methods**

Exclusions related to liver morbidity: hepatocellular carcinoma, alcoholic liver disease, hemochromatosis, autoimmune hepatitis, chronic viral hepatitis B or C, any diagnosis of cirrhosis (including primary or secondary biliary and alcoholic), primary biliary or sclerosing cholangitis, Wilson’s disease, alpha1 antitrypsin deficiency) at any time prior to diagnosis of NAFLD/NASH, a record of drug-induced liver toxicity or of pregnancy-related liver disease including acute fatty liver of pregnancy, eclampsia, hyperemesis gravidarum, intrahepatic cholestasis and HELLP (haemolysis, elevated liver enzymes and low platelets) syndrome

## Variables

We extracted the following clinical data if recorded between 2 years prior to and 6 months after index date: body mass index (BMI) serum alanine transaminase (ALT), aspartate transaminase (AST), albumin, platelet count, systolic blood pressure and statin use. When several measurements were available, the measurement closest to index date was used. Smoking was “current” if the patient smoked up to 5 years prior to index date or anytime post index date, and “non-current” otherwise. History of diabetes and hypertension were defined as a record occurring any time prior to or at index date.

Code lists for all clinical diagnoses (exclusion criteria, exposure, covariates and events of interest) were generated using a semantic harmonization process that involved mapping clinical diagnoses in each terminology to interchangeable concepts coded using the Unified Medical Language System (UMLS) concepts, thereby ensuring comparability between databases [18].

## Data analysis

Laboratory measurements that were greater than the database-specific mean plus 3 times the standard deviation were excluded as outliers. Values of aspartate transaminase (AST), alanine transaminase (ALT) and platelet counts below 5, and values of body mass index (BMI) <15 or >60 kg/m^2^ were also excluded. We calculated the Fib-4 non-invasive liver fibrosis score for patients with data available on age, AST, ALT, and platelets counts using the following equation [19]: Fib-4 = $Age\left( years \right]\times AST[U/L])/(platelet\left[ {10}^{8} \right]\times\surd ALT[U/L])$ and used cut-off values of 1.30 and 2.67 for low and high risk respectively. Values 1.30-2.67 indicated indeterminate risk of fibrosis.

Analyses were performed separately for each of the four databases. Descriptive statistics were summarised using percentages for categorical variables, mean and standard deviation for normally distributed variables, and median and interquartile range for skewed variables. Incidence rates of non-alcoholic cirrhosis and hepatocellular carcinoma were estimated within each group by dividing the number of incident events divided by the total number of person-years at risk. Ninety-five % confidence intervals for incidence rates were estimated assuming a Poisson distribution. Hazard ratios for the risk of incident cirrhosis or hepatocellular carcinoma were estimated using Cox proportional hazard models. The models were stratified by matching pair and adjusted for age, smoking status and BMI. As BMI was not available in all patients, analyses were run in the entire database and in the subset of patients with BMI data available, adjusting for sex and smoking only, to evaluate the confounding effect of BMI. Hazard ratios (HR) were pooled across databases by random-effects meta-analysis. Heterogeneity across databases was tested using chi squared test on the Q statistic, and by providing the *I*^2^ statistic which gives the percentage of variation among studies that is due to heterogeneity across databases, rather than to variation among individual patients within a database. Hazard ratios were estimated by subgroups according to gender, BMI (obese: BMI≥30kg/m^2^, versus non-obese: BMI<30kg/m^2^), smoking status, age group (<55 years old versus ≥55 years old), hypertension status, and diabetes status, by fitting an interaction term between NAFLD/NASH exposure and the subgroup of interest. To compare the associations between history of diabetes, BMI, hypertension, smoking status with incident liver outcomes (earliest of cirrhosis or HCC) between NAFLD/NASH patients and controls, we fitted a Cox model including these covariates stratified by database and by matching pair in NAFLD/NASH and matched controls separately.

**Table S1:** Attrition table showing patients with recorded diagnoses of NAFLD or NASH and matched unexposed controls.

| Database | HSD  (Italy) | IPCI  (The Netherlands) | SIDIAP  (Spain) | THIN  (UK) | Total |
| --- | --- | --- | --- | --- | --- |
| Number ever registered in database at 31/12/2015 | 1,571,651 | 2,225,925 | 5,488,397 | 14,701,817 | 23,987,790 |
| Number of individuals with at least 1 year since registration in database | 1,544,573 | 1,780,500 | 5,259,575 | 10,197,633 | 18,782,281 |
| Number of NAFLD/NASH patients* | NAFLD/NASH: 22,424 | NAFLD/NASH: 18,432 | NAFLD only: 73,045  NASH only: 1,816 | NAFLD only: 20,424  NASH only: 896 | NAFLD/NASH:  136,703  NAFLD only:  93,469  NASH only:  2,712 |
| Number of matched unexposed patients (ratio unexposed / exposed) | Matched to:  NAFLD/NASH: 1,760,522  (79) | Matched to: NAFLD/NASH: 1,732,990  (94) | Matched to:  NAFLD/NASH: 5,376,254  (72)  NAFLD only: 5,263,189  (72)  NASH only: 128,721  (71) | Matched to:  NAFLD/NASH: 2,386,503 (113)  NAFLD only: 2,293,469 (112)  NASH only: 112,731 (125) | NAFLD/NASH:  11,256,269  NAFLD only:  7,556,658  NASH:241,452 |

*after excluding concomitant liver disease and recorded excess alcohol consumption

**Table S2:** Descriptive characteristics of coded NAFLD or NASH patients and matched unexposed cohorts in SIDIAP and THIN

| Baseline characteristics | SIDIAP - Spain | | THIN - UK | | SIDIAP - Spain | | THIN - UK | |
| --- | --- | --- | --- | --- | --- | --- | --- | --- |
|  | NAFLD only | Matched non- NAFLD | NAFLD only | Matched non- NAFLD | NASH only | Matched non- NASH | NASH only | Matched non- NASH |
| Follow-up in years prior to index date: Median (IQR) | 5.3 (3.2-7.1) | 5.3 (3.2- 7.1) | 13.5 (5.5 -23.4) | 13.8 (5.9-23.4) | 0.6 (0.3 - 1.1) | 0.6 (0.3 - 1.2) | 12.0 (5.1 - 21.8) | 13.3 (5.7 - 22.6) |
| Follow-up in years post index date: Median (IQR) | 3.4 (1.7-5.5) | 3.4 (1.7-5.5) | 3.0 (1.3-5.8) | 3.0 (1.3 - 5.7) | 8.3 (7.1-8.7) | 8.3 (7.3-8.7) | 4.1 (1.9-7.4) | 3.8 (1.6-6.7) |
| Age in years, mean (SD) | 56.0 (13.3) | 54.6 (13.0) | 54.4 (13.4) | 53.5 (13.5) | 52.8 (15.3) | 50.9 (14.5) | 52.8 (15.3) | 53.6 (13.3) |
| Gender, % of Males | 52.5% | 48.7% | 50.2% | 50.9% | 54.6% | 52.1% | 55.4% | 55.8% |
| Current smokers, % | 17.5% | 15.4% | 16.8% | 17.9% | 19.3% | 15.2% | 15.0% | 18.6% |
| History of Type 2 diabetes, % | 20.2% | 10.5% | 21.4% | 7.1% | 10.7% | 8.2% | 26.5% | 7.0% |
| History of hypertension, % | 43.0% | 29.4% | 41.2% | 26.1% | 29.3% | 22.9% | 42.6% | 25.9% |
| Aspartate transaminase *(IU/L), median (IQR) | 29 (22-41) | 21 (18-27) | 31 (23-45) | 22 (19-27) | 31 (23-45) | 21 (17-26) | 42 (28-65) | 23 (19-28) |
| Alanine transaminase *(IU/L), median (IQR) | 35 (22-55) | 20 (15-28) | 43 (27-66) | 23 (17-31) | 39 (24-62) | 21 (17-26) | 50.5 (31-82) | 23 (17-33) |
| FIB4 score*, |  |  |  |  |  |  |  |  |
| Low risk (FIB4 <1.30) | 64.2% | 65.8% | 63.1% | 62.8% | 62.2% | 72.9% | 47.0% | 63.0% |
| Indeterminate risk (FIB4: 1.30-2.67) | 30.0% | 31.0% | 25.7% | 26.7% | 27.3% | 25.7% | 37.0% | 26.1% |
| High Risk (FIB4>2.67) | 5.4% | 3.2% | 11.2% | 10.5% | 10.6% | 2.5% | 16.0% | 10.9% |

**Table S3:** Number of patients with data available in coded NAFLD/NASH and matched unexposed cohorts

| Number (%) of patients with data on: | HSD - Italy | | IPCI - The Netherlands | | SIDIAP - Spain | | THIN - UK | | Total population | |
| --- | --- | --- | --- | --- | --- | --- | --- | --- | --- | --- |
|  | NAFLD/NASH | Matched control | NAFLD/NASH | Matched control | NAFLD/NASH | Matched control | NAFLD/NASH | Matched control | NAFLD/  NASH | Matched control |
| Entire sample | 22,424 | 1,760,522 | 18,432 | 1,732,990 | 74,678 | 5,376,254 | 21,169 | 2,386,503 | 136,703 | 11,256,269 |
| BMI | 11,275 | 597,849 | 8,359 | 495,498 | 52,255 | 2,791,764 | 16,464 | 1,278,909 | 88,353 | 5,164,020 |
| AST | 15,227 | 729,982 | 7,543 | 197,998 | 50,520 | 1,766,227 | 4,385 | 240,003 | 77,675 | 2,934,210 |
| ALT | 16,153 | 790,374 | 12,385 | 506,743 | 64,477 | 3,523,348 | 16,738 | 1,091,276 | 109,753 | 5,911,741 |
| FIB4 score | 9,948 | 497,738 | 4,580 | 115,588 | 46,785 | 1,595,548 | 2,658 | 115,322 | 63,971 | 2,324,196 |
| AST to ALT ratio | 14,915 | 718,317 | 7,258 | 187,971 | 48,994 | 1,700,198 | 3,058 | 141,218 | 74,225 | 2,747,704 |
| BARD score | 8,280 | 337,723 | 3,690 | 79,826 | 34,980 | 1,059,648 | 2,531 | 103,255 | 49,481 | 1,580,452 |

BMI: Body Mass Index; AST: aspartate transaminase; ALT: alanine transaminase

**Table S4:** Incidence rate of liver outcomes in four primary care databases

| Database | Event of interest | Sample | Number of  person-years | Number of events | Incidence rate (95% CI)  per 1,000 person-years |
| --- | --- | --- | --- | --- | --- |
| Events in NAFLD/NASH and matched non NAFLD/NASH patients | | | | | |
| HSD | Cirrhosis | NAFLD/NASH | 125584 | 58 | 0.46 (0.35; 0.6) |
|  | Cirrhosis | Matched control | 9743461 | 1477 | 0.15 (0.14; 0.16) |
|  | Hepatocellular carcinoma | NAFLD/NASH | 125713 | 36 | 0.29 (0.2; 0.4) |
|  | Hepatocellular carcinoma | Matched control | 9745522 | 1167 | 0.12 (0.11; 0.13) |
| IPCI | Cirrhosis | NAFLD/NASH | 40349 | 87 | 2.16 (1.73; 2.66) |
|  | Cirrhosis | Matched control | 3875430 | 1027 | 0.27 (0.25; 0.28) |
|  | Hepatocellular carcinoma | NAFLD/NASH | 40449 | 15 | 0.37 (0.21; 0.61) |
|  | Hepatocellular carcinoma | Matched control | 3876575 | 222 | 0.06 (0.05; 0.07) |
| SIDIAP | Cirrhosis | NAFLD/NASH | 277987 | 211 | 0.76 (0.66; 0.87) |
|  | Cirrhosis | Matched control | 20100000 | 3401 | 0.17 (0.16; 0.17) |
|  | Hepatocellular carcinoma | NAFLD/NASH | 278370 | 73 | 0.26 (0.21; 0.33) |
|  | Hepatocellular carcinoma | Matched control | 20100000 | 1899 | 0.09 (0.09; 0.1) |
| THIN | Cirrhosis | NAFLD/NASH | 86329 | 200 | 2.32 (2.01; 2.66) |
|  | Cirrhosis | Matched control | 9623809 | 1438 | 0.15 (0.14; 0.16) |
|  | Hepatocellular carcinoma | NAFLD/NASH | 86687 | 52 | 0.6 (0.45; 0.79) |
|  | Hepatocellular carcinoma | Matched control | 9625487 | 804 | 0.08 (0.08; 0.09) |
| Events in NASH only and matched non-NASH only patients | | | | | |
| SIDIAP | Cirrhosis | NASH | 13427 | 38 | 2.83 (2; 3.88) |
|  | Cirrhosis | non-NASH | 987702 | 188 | 0.19 (0.16; 0.22) |
|  | Hepatocellular carcinoma | NASH | 13524 | 15 | 1.11 (0.62; 1.83) |
|  | Hepatocellular carcinoma | non-NASH | 988039 | 114 | 0.12 (0.1; 0.14) |
| THIN | Cirrhosis | NASH | 4472 | 26 | 5.81 (3.8; 8.52) |
|  | Cirrhosis | non-NASH | 532596 | 60 | 0.11 (0.09; 0.15) |
|  | Hepatocellular carcinoma | NASH | 4539 | 6 | 1.32 (0.49; 2.88) |
|  | Hepatocellular carcinoma | non-NASH | 532639 | 35 | 0.07 (0.05; 0.09) |
| Events in NAFLD only and matched non-NAFLD only patients | | | | | |
| SIDIAP | NASH | NAFLD | 265672 | 7 | 0.03 (0.01; 0.05) |
|  | NASH | non-NAFLD | 1.92E+07 | 160 | 0.01 (0.01; 0.01) |
|  | Cirrhosis | NAFLD | 265358 | 174 | 0.66 (0.56; 0.76) |
|  | Cirrhosis | non-NAFLD | 1.92E+07 | 3246 | 0.17 (0.16; 0.17) |
|  | Hepatocellular carcinoma | NAFLD | 265647 | 58 | 0.22 (0.17; 0.28) |
|  | Hepatocellular carcinoma | non-NAFLD | 1.92E+07 | 1766 | 0.09 (0.09; 0.1) |
| THIN | NASH | NAFLD | 79,654 | 103 | 1.29 (1.06; 1.57) |
|  | NASH | non-NAFLD | 8,861,316 | 463 | 0.05 (0.05; 0.06) |
|  | Cirrhosis | NAFLD | 82516 | 179 | 2.17 (1.86; 2.51) |
|  | Cirrhosis | non-NAFLD | 9174103 | 1250 | 0.14 (0.13; 0.14) |
|  | Hepatocellular carcinoma | NAFLD | 82816 | 47 | 0.57 (0.42; 0.75) |
|  | Hepatocellular carcinoma | non-NAFLD | 9175634 | 759 | 0.08 (0.08; 0.09) |

**Table S5:** Descriptive characteristics of coded NAFLD/NASH patients and matched non-NAFLD/NASH in sample of patients with and without BMI data available, all datasets combined

| Baseline characteristics | With BMI data | | Without BMI data | |
| --- | --- | --- | --- | --- |
|  | NAFLD/NASH | Matched control | NAFLD/NASH | Matched control |
| Number of individuals | 88,353 | 5,164,020 | 48,350 | 6,092,249 |
| Age in years, mean (SD) | 57.2 (13.3) | 57.8 (12.7) | 53.4 (13.9) | 51.9 (13.3) |
| Gender, % of Males | 50.3% | 46.1% | 56.6% | 53.6% |
| Current smokers, % | 19.5% | 20.2% | 11.2% | 9.7% |
| History of Type 2 diabetes, % | 26.9% | 17.3% | 7.0% | 3.2% |
| History of hypertension, % | 50.1% | 42.8% | 27.9% | 17.7% |
| Alanine transaminase (IU/L), median (IQR)* | 34 (22-54) | 21 (16-29) | 38 (23-55) | 21 (16-29) |
| Aspartate transaminase (IU/L), median (IQR)* | 28 (21-39) | 21 (18-26) | 28 (21-38) | 21 (18-26) |
| FIB 4 score*  Low risk (<1.30)  Indeterminate (1.30-2.67)  High (>2.67) | 62.9%  31.8%  5.3% | 62.4%  33.9%  3.6% | 71.0%  25.4%  3.6% | 70.0%  27.5%  2.4% |

*In subsets of patients with data available.

**Table S6:** Median and interquartile range (in years) for time to event in coded NAFLD and matched non-NAFLD who experience a cirrhosis or hepatocellular carcinoma event during follow-up

| Patient group | Non-alcoholic steatohepatitis | | | Cirrhosis | | | Hepatocellular carcinoma | | |
| --- | --- | --- | --- | --- | --- | --- | --- | --- | --- |
|  | Events | Events <6  months after index date | Median time to event (IQR) | Events | Events <6  months after index date | Median time to event (IQR) | Events | Events <6  months after index date | Median time to event (IQR) |
| **HSD database** | | | | | | | | | |
| NAFLD/NASH | - | - | - | 58 | 10 | 3.3 (0.9-5.9) | 36 | 2 | 2.6 (0.6-7.1) |
| **IPCI database** | | | | | | | | | |
| NAFLD/NASH | - | - | - | 87 | 40 | 0.6 (0.1-1.6) | 15 | 4 | 0.5 (0.2-3.2) |
| **SIDIAP database** | | | | | | | | | |
| NAFLD/NASH | - | - | - | 211 | 21 | 2.9 (1.3-4.6) | 73 | 3 | 2.9 (1.0-4.6) |
| NAFLD | 7 | 6 | 0.22 (0.04-0.35) | 174 | 17 | 2.9 (1.4-4.5) | 58 | 1 | 2.9 (1.2-4.6) |
| NASH | - | - | - | 38 | 4 | 3.0 (1.2-5.3) | 15 | 2 | 1.9 (0.3-5.3) |
| **THIN database** | | | | | | | | | |
| NAFLD/NASH | - | - | - | 200 | 51 | 1.7 (0.5-4.5) | 52 | 4 | 4.1 (1.2-8.2) |
| NAFLD | 103 | 32 | 2.8 (0.9-7.7) | 179 | 46 | 1.9 (0.5; 4.6) | 47 | 4 | 3.1 (1.0; 8.2) |
| NASH | - | - | - | 26 | 8 | 0.9 (0.5-2.6) | 6 | 0 | 6.7 (4.9-8.2) |

**Figure S1.** Subgroup analysis of the association between coded NAFLD/NASH and incident (A) cirrhosis and (B) hepatocellular carcinoma events by medical history and demographics

**Figure S2** Hazard ratio (HR)for Cirrhosis (A) Adjusted for age and smoking in all patients and (B) Adjusted for age and smoking in patients with BMI

**Figure S3** Hazard ratio (HR) for HCC (A) Adjusted for age and smoking in all patients and (B) Adjusted for age and smoking in patients with BMI

**Figure S4** Fib-4 Association with (A) cirrhosis or (B) HCC

A

B

**Figure S5** Risk of coded NASH in patients with coded NAFLD
